# Public health unit engagement in school mental health programs and adolescent mental health during the COVID-19 pandemic: COMPASS, 2018–2022

**DOI:** 10.1093/pubmed/fdae179

**Published:** 2024-08-08

**Authors:** Claire Benny, Brendan T Smith, Karen A Patte, Scott T Leatherdale, Roman Pabayo

**Affiliations:** School of Public Health, University of Alberta, Edmonton, AB T6G 1C9, Canada; Department of Health Promotion, Chronic Disease, and Injury Prevention, Public Health Ontario, Toronto, ON M5B 1W2, Canada; Department of Health Promotion, Chronic Disease, and Injury Prevention, Public Health Ontario, Toronto, ON M5B 1W2, Canada; Dalla Lana School of Public Health, University of Toronto, Toronto, ON M5T 3M7, Canada; Department of Health Sciences, Brock University, St. Catharines, ON LS2 3A1, Canada; School of Public Health Sciences, University of Waterloo, Waterloo, ON N2L 3G1, Canada; School of Public Health, University of Alberta, Edmonton, AB T6G 1C9, Canada; School of Public Health, University of Alberta, Centre for Healthy Communities, Edmonton, AB T6G 2R3, Canada

**Keywords:** COVID-19, mental health, public health

## Abstract

**Background:**

Public health unit (PHU) engagement in schools is important for promoting wellness in students. We aimed to investigate if PHU engagement with schools may have provided protection against the risk of depression and anxiety in students during the COVID-19 pandemic.

**Methods:**

We used longitudinal data from the Cannabis, Obesity, Mental health, Physical activity, Alcohol use, Smoking and Sedentary behaviour survey between the 2018/19 and 2020/21 academic years. Multilevel models were used to assess the association between PHU engagement with school mental health programs prior to the COVID-19 pandemic and depressive (Center for Epidemiologic Studies Depression scale Revised) and anxiety symptoms (Generalized Anxiety Disorder scale) during the COVID-19 pandemic.

**Results:**

The sample included 23 894 students across 104 secondary schools in British Columbia, Alberta, Ontario and Quebec. In confounder-adjusted models, PHU engagement before the pandemic was not associated with student depressive symptoms (B = −0.01, 95% CI = −0.04, 0.02), but was protective against anxiety symptoms (B = −0.03, −0.06, 0.001) during the COVID-19 pandemic.

**Discussion:**

The results highlight that PHU engagement with mental health programming in schools was protective against anxiety for students during the COVID-19 pandemic. The findings support the importance of PHU engagement for improving student mental health and pandemic recovery.

## Introduction

Depressive and anxiety symptoms among teens increased markedly during the COVID-19 pandemic.^1^ Schools represent an important space for early intervention of mental health concerns as the location where young people spend most of their waking hours on weekdays. In Canada, public health units (PHUs), health regions and other similar jurisdictions are responsible for implementing public health programs across provinces and territories. These units are vital to improving and protecting population health. As indicated by a systematic review and meta-analysis, for every $1 spent on public health, governments can save $14 on average over a decade,^2^ yielding a large financial return. Despite this, there have been recent movements to reduce funding and change the structure of PHUs within some Canadian provinces.^3^ During the COVID-19 pandemic, public health funding was largely allocated toward infectious disease efforts, leaving a critical gap in schools where students were suffering indirect consequences of the pandemic (i.e. mental health problems).

The effects of adverse mental health during adolescence are pervasive throughout the lifespan.^4^ Youth may experience issues in coping with adverse mental health, due to emotional maturity and limited life experience. In adolescence, healthy coping mechanisms and mental health care seeking behaviours are less frequent than in adults,^5^ which may lead to continued mental health problems or the use of substances to self-medicate.^6^^,^^7^ Navigating mental health in youth may be especially difficult, because the hormonal fluctuations associated with puberty often result in negative affect and angst, which resemble some symptoms of mental disorders.^8^ As such, mental health conditions may be less detectable among youth. In addition, the stigma associated with seeking help for mental health care is also highly prevalent in school environments.^5^^,^^9^ As a result, a large proportion youth with mental health conditions go untreated or undetected.^4^^,^^10^

Most mental health conditions among youth go untreated or undetected and the effects of poor mental health are long-lasting. Complicated emotions associated with mental health problems may manifest in feelings of mistrust, isolation and disorder. These feelings may be more common in times of social and economic uncertainty, such as in the COVID-19 pandemic.^11^^,^^12^ Based on the existing literature, it is clear that these feelings can further worsen mental health^13^ and research highlights that the effects of COVID-19 were more harmful for mental health for girls compared to boys.^12^ In addition, because youth are still developing, untreated mental health issues in youth may lead to issues with social, emotional and even physical development.^4^^,^^14^ This is important because problems in youth can lead to resounding issues throughout the lifespan and into adulthood, causing a larger and longer health burden on the individual and the health care system.

PHUs represent a critical point of engagement with schools, as they can provide additional supports for students by improving and introducing programs to improve student health. Schools that report joint program development with local PHUs are more likely to have students with better mental health and students that engage in fewer bullying behaviours^15^; albeit, this evidence is cross-sectional and does not highlight any temporal associations between general public health engagement and mental health. Programs developed or implemented in joint capacity between schools and PHUs may present a means for improving student health. Specifically, existing studies have shown that in schools where PHUs collaborate on substance use programs, the prevalence of substance use is reduced.^16–18^ It is possible that when PHUs engage with schools in managing student mental health, there are benefits. Given that student mental health may have worsened during the pandemic, and that public health engagement with schools has been associated with better health,^15^^,^^19^ we aimed to identify if students attending schools where PHUs were engaged in mental health programs during the COVID-19 pandemic have experienced lover risk of depression and anxiety.

## Methods

### Sample

The current longitudinal study sample was taken from the Cannabis, Obesity, Mental health, Physical activity, Alcohol use, Smoking and Sedentary behaviour (COMPASS) survey, which is a prospective cohort study (2012–present), designed to annually collect multilevel longitudinal survey data from students and secondary schools across British Columbia, Alberta, Ontario and Quebec.^20^ All students attending participating schools were eligible to participate and were recruited using an active-information passive parental consent protocol. Student data is linked across years of participation using a self-generated anonymous code. In the current study, we used data collected in the 2018/19 academic year and included any student who had responded at least once in the follow-up period of 2019/20, 2020/21 or 2021/22 survey waves. Graduating students in 2018/19 were not included in our sample as they would not be followed up over the remaining study period. COMPASS involves an in-class student questionnaire, which was initially a machine-readable paper-based survey and transitioned to an online platform immediately following the onset of COVID-19. The sample of our study included 23,894 students across 104 schools across Canada. Student data were linked to school-level data from the COMPASS School Policies and Procedures (SPP) module. The SPP is an online survey completed annually by school administrators most familiar with the school health environment. All procedures received ethics approval from the University of Waterloo (ORE#30118), Brock University (REB#18-099), University of Alberta (#RES0050375), CIUSSS de la Capitale-Nationale–Université Laval (#MP-13-2017-1264) and participating school boards.

### Measures

#### Outcomes: depressive and anxiety symptoms

Depressive and anxiety scores were calculated based on the 10-item Center for Epidemiologic Studies Depression scale Revised (CES-D) and the 7-item Generalized Anxiety Disorder scale (GAD-7), respectively. These scales have demonstrated validity in adolescents^21–23^ and measurement invariance by gender specifically in the COMPASS survey.^24^ Depressive scores were measured based on symptoms reported in the past week, whereas anxiety scores were measured based on how often students experienced each symptom in the past 2 weeks.^25^ Measures of depression and anxiety were standardized using the z-transformation.

#### Exposure: public health engagement with school mental health programs

PHU engagement with school mental health programs was measured using the SPP for the 2020–21 sample, as schools at this point would be in the midst of the COVID-19 pandemic. For the SPP, school administrators reported on engagement with mental health programs by the PHU in the past twelve months. Engagement was described on four levels: (1) ‘no contact with local public health unit’; (2) ‘provided information/resources/programs’ such as print materials and toolkits; (3) ‘solved problems jointly’; and (4) ‘developed and implemented program activities jointly’. Schools were coded as either having no engagement in the past 12 months (option 1) or having at least some engagement (options 2–4). Further analysis modeled the association between each type of public health engagement (providing information/resources/programs, solving problems jointly and developed/implemented program activities jointly) and mental health scores in COVID-19.

#### Time points

In March 2020, the World Health Organization announced the onset of the COVID-19 pandemic. Those observations recorded prior to the March 2020 school closures associated with COVID-19 were coded as ‘pre-COVID-19’ [time(T)1], and those following school closures and in the 2019/20 wave were coded as ‘peri-COVID-19’ (i.e. ‘during’ COVID-19; T2) thereafter in years 2020/21 (T3) and 2021/22 (T4). Participants were included if they had responded at T1 and at least once during the COVID-19 pandemic (T2–T4).

#### Covariates

At the individual level the models adjusted for age (in years), gender (boy, girl for 2018/19 and boy, girl or ‘other gender identity, or not reported’ for 2019/20–2021/2), race/ethnicity [Black, Hispanic, Asian or other (selected ‘other’, multiple responses, or Métis, First Nations or Inuit as ethics restrictions precluded the identification of students with Indigenous heritage for separate study^26^)] and year of study. In the COMPASS survey, students were asked to identify themselves as ‘male or female’ in T1, likely capturing elements of both biological sex and gender as a social construct. We have presented our findings using the term gender, consistent with existing studies.^27^^,^^28^ At the school level, the models adjusted for the type of school (i.e. public vs. private school).

### Statistical analysis

Two level growth curve models were used to examine the research question.^29^ Initially, null models were conducted to calculate the intraclass correlation coefficient (ICC), or the degree of variation at each level of our models. Next, unadjusted models were carried out to determine the crude association between public health engagement in school mental health programs before the pandemic and student mental health scores over time during the pandemic. Finally, fully adjusted models including both individual-level and school-level covariates were run to see the association between public health engagement before the pandemic in school mental health programs and student mental health scores over time. This final model was the most appropriate model and the most reflective of the real association between public health engagement in schools and mental health scores during the COVID-19 pandemic. Analyses were repeated for each type of public health engagement.

## Results

Our sample included 23 894 students from 104 schools across British Columbia, Alberta, Ontario and Quebec. The majority of students self-reported as white (76.9%) and as girls (54.5%). Schools were mostly public institutions (92.3%), and 54.2% reported PHU engagement over the study period. Further details are available in Table 1. The ICC for depressive scores was 0.49 between students (95% CI: 0.48, 0.50), and 0.02 between schools over time (95% CI: 0.01, 0.03). The ICC for anxiety scores were 0.52 (95% CI: 0.51, 0.53) and 0.03 (95% CI: 0.02, 0.04), respectively.

**Table 1:** Distribution of characteristics of the study sample at baseline, COMPASS 2018–19

Individual-level characteristics *n* %			
**Gender** ^**a**^	Girl	13 041	54.5
	Boy	10 853	45.5
Race/ethnicity	White	18 343	76.9
	Black	721	3.0
	Asian	1883	7.9
	Latin American	654	2.7
	Other	2262	9.5
		**Mean**	**SD**
Age	In years	14.2	0.1
School-level characteristics		***N***	%
	Private	8	7.7
	Public	96	92.3

^a^Other gender identity was not recorded at baseline.

Unadjusted models indicated that in schools where PHUs were engaged with mental health programs before COVID-19, students reported no difference in depressive scores (B = −0.01, 95% CI: −0.03, 0.02, Table 2) during the follow-up, whereas students did report lower anxiety scores (B = −0.05, 95% CI: −0.07, −0.02, Table 3) following the onset of COVID-19. Results were consistent when adjusting for covariates, with a slight attenuation of the protective effect on anxiety symptoms (B = −0.03, −0.06, 0.001, Table 3).

**Table 2:** Unadjusted and adjusted association between PHU engagement with school mental health programs and student depressive scores during the COVID-19 pandemic, COMPASS 2018–2021

		Unadjusted models			Adjusted models		
		Coef	95% CI	*P* value	Coef	95% CI	*P* value
Public health unit (PHU) engagement with school mental health programs (ref: none)		-0.03	−0.09, 0.03	0.366	0.01	−0.01, 0.04	0.403
Peri-COVID-19 (ref: pre-COVID-19)		0.34	0.32, 0.36	< 0.001	0.11	0.07, 0.14	<0.001
PHU engagement with school mental health programs × Peri-COVID-19		−0.01	−0.03, 0.02	0.667	−0.01	−0.04, 0.02	0.468
Age	In years				0.08	0.07, 0.09	<0.001
Gender (ref: girl)	Boy				−0.46	−0.48, 0.44	<0.001
	Other gender identity, or not reported				0.38	0.29, 0.47	<0.001
Race/ethnicity (ref: White)	Black				0.07	0.01, 0.13	0.026
	Asian				0.10	0.06, 0.14	<0.001
	Latin American				0.15	0.09, 0.21	<0.001
	Other race/ethnicity				0.18	0.14, 0.21	<0.001
School type (ref: public)	Private				0.44	−0.46, 1.34	0.341
Year of study	in years				0.04	0.022, 0.06	<0.001

**Table 3:** Unadjusted and adjusted association between PHU engagement with school mental health programs and student anxiety scores during the COVID-19 pandemic, COMPASS 2018–2021

		Unadjusted model			Adjusted model		
		Coef	95% CI	*P* value	Coef	95% CI	*P* value
Public health unit (PHU) engagement with school mental health programs (ref: none)		−0.03	−0.11, 0.04	0.400	0.02	0.00, 0.05	0.057
Peri-COVID-19 (ref: pre-COVID-19)		0.37	0.35, 0.38	< 0.001	0.03	0.00, 0.06	0.063
PHU engagement with school mental health programs × Peri-COVID-19		−0.05	−0.07, −0.02	< 0.001	−0.03	−0.06, 0.00	0.043
Age	In years				0.10	0.09, 0.11	< 0.001
Gender (ref: girl)	Boy				−0.53	−0.55, −0.05	< 0.001
	Other gender identity, or not reported				0.19	0.11, 0.28	< 0.001
Race/Ethnicity (ref: White)	Black				−0.03	−0.09, 0.03	0.331
	Asian				0.02	−0.02, 0.05	0.384
	Latin American				0.11	0.05, 0.17	< 0.001
	Other race/ethnicity				0.14	0.11, 0.17	< 0.001
School type (ref: public)	Private				0.52	−0.35, 1.39	0.242
Year of study	in years				0.05	0.03, 0.07	< 0.001

Consistently, PHU engagement involving providing information/resources/programs (e.g. posters, toolkits) before the pandemic was not associated with depressive symptoms (B = −0.017, 95% CI: −0.05, 0.01) in adjusted analyses, but was protective against anxiety symptoms (B = −0.03, 95% CI: −0.06, −0.001) during the COVID-19 pandemic. Similarly, PHU engagement involving solving problems jointly with schools before the pandemic was not associated with depressive symptoms (B = 0.03, 95% CI: −0.02, 0.08), but was protective against anxiety symptoms during COVID-19 (B = −0.06, 95% CI: −0.11, −0.01) in adjusted analyses. Conversely, PHU engagement involving developing and implementing programs jointly with schools before the pandemic was associated with increased depressive scores (B = 0.10, 95% CI: 0.02, 0.18), but was not associated with anxiety scores during COVID-19 (B = 0.04, 95% CI: −0.04, 0.11) in adjusted analyses. See additional results in Table 4.

**Table 4:** Regression coefficients detailing the association between type of PHU engagement in school mental health programs and student depression and anxiety scores during the COVID-19 pandemic, COMPASS 2018–21

	Depression scores						Anxiety scores					
	Unadjusted			Adjusted^a^			Unadjusted			Adjusted		
Level of PHU-engagement	Coef	95% CI	*P* value	Coef	95% CI	*P* value	Coef	95% CI	*P* value	Coef	95% CI	*P* value
Provided information/resources/programs (e.g. posters, toolkits)	−0.004	−0.03, 0.02	0.77	−0.017	−0.05, 0.01	0.256	−0.02	−0.05, 0.00	0.089	−0.03	−0.06, 0.00	0.044
Solved problems jointly	0.002	−0.04, 0.05	0.925	0.03	−0.02, 0.08	0.283	−0.08	−0.13, 0.03	0.001	−0.06	−0.11, −0.01	0.011
Developed/implemented program activities jointly	0.099	0.02, 0.18	0.015	0.11	0.03, 0.20	0.009	0.02	−0.05, 0.10	0.536	0.04	−0.04, 0.11	0.367

^a^Adjusted for age, gender, race/ethnicity, year of study and type of school.

## Discussion

### Main findings

The results of this study indicate that PHU involvement in school mental health programs following the onset of COVID-19, compared to before COVID-19, was associated with reductions in anxiety symptoms. Specifically, by the type of PHU involvement, solving programs jointly was beneficial for student anxiety. No associations were found between PHU engagement in school mental health programs during COVID-19 and student depressive scores. However, it should be noted this null result could be because of less pronounced changes in depressive symptoms during the COVID-19 pandemic when compared to anxiety symptoms^30^ and that in cases of comorbidity, anxiety symptoms generally precede depressive symptoms.^31^ Evidence from an international study suggest that countries that swiftly enforced strict public health measures actually experienced lower levels of depression compared to those that were slower to implement such measures.^32^ As the Canadian government quickly enacted public health countermeasures, this may explain the null association between public health engagement and depressive scores during the COVID-19 pandemic.

Results were less consistent when examined by type of public health engagement with schools. Specifically, in schools where PHUs were engaged in developing and implementing programs jointly, students reported an overall increase in depressive scores following the onset of COVID-19. Conversely, in schools where PHUs were engaged in solving problems jointly, students reported overall decreases in anxiety scores following the onset of COVID-19. It may be that some methods of engagement are better or worse at either promoting mental health and/or preventing mental illness than others. The effectiveness and quality of these programs for improving mental health and the level of engagement of PHUs was not measured in our study.

### What is already known

The mental health of adolescents has deteriorated since the beginning of the COVID-19 pandemic. This decline may potentially be attributed not only to concerns about the virus but also to the isolation and social separation caused by public health measures like school closures, food insecurity and anxiousness about the future. The findings imply that partnerships between schools and PHUs could be an important factor in improving adolescent mental health in recovery from the COVID-19 pandemic. Additional support for schools from PHUs may include joint development of counselling programs and school mental health awareness campaigns, amongst other interventions. Introducing relationships between PHUs and schools in spaces where those relationships are currently limited may also act as a facilitator for improving student health.

### What this study adds

There are several strengths of this study, including that we used data from a large number of schools and students to address the research question. The study used well-established scales for assessing depression and anxiety (specifically, the CES-D and GAD-7) and involved secondary school students from four Canadian provinces.^33^ While it is worth noting that one survey wave in 2019/20 had a lower response rate due to the emergence of COVID-19 and the transition to online learning and survey distribution methods, the use of data following this initial disruption improves the study quality. Findings from this study strengthen the knowledge on the association between public health and mental health, given that depressive and anxiety scores were collected both prior to and peri-pandemic. This improves upon existing studies that retrospectively measured pre-exposure by asking students how their mental health was during compared to before the COVID-19 pandemic.^34–37^ The measurements used in this study minimize the potential for recency and other reporting biases. Moreover, the associations between PHU involvement and youth mental health during the COVID-19 pandemic have not yet been studied.

### Limitations

The findings of this study should be interpreted given certain limitations. Firstly, it is important to acknowledge that COMPASS, while involving numerous schools in rural to large urban areas in British Columbia, Alberta, Ontario and Quebec, employs convenience sampling techniques. Therefore, the results may not be readily generalizable to larger populations, although they may still be applicable to students attending schools with similar characteristics. There is also a possibility of reporting bias in COMPASS, as social desirability bias might affect how youth self-report their mental health. Additionally, conducting questionnaires online due to school closures could introduce some bias, but controlling for pre- versus peri-pandemic responses likely mitigates some of this bias. Another study limitation is the potential for residual confounding. It is possible that there are other factors not considered in the analysis that could explain some of the variations in the association between public health engagement and adolescent depression and anxiety. For example, household income, which was not collected, could be a residual confounder. Furthermore, school funding per number of students was not available and may influence the association between PHU funding and mental health in the COVID-19 pandemic.

Researchers looking to further this line of inquiry may continue to track trends in student mental health in recovery from the COVID-19 pandemic. It also may be of interest to use quasi-experimental designs to improve the strength of this evidence. For example, interrupted time series techniques may be helpful for inferring change immediately resulting from the COVID-19 pandemic once further time points become available. Overall, this study highlights the pivotal role of PHUs in mitigating the adverse effects of the COVID-19 pandemic on adolescent mental health, particularly in reducing anxiety symptoms through solving problems jointly. Thus, this study highlights the importance of proactive public health interventions in safeguarding mental well-being during challenging times like the COVID-19 pandemic.

## Data Availability

The data underlying this article will be shared on reasonable request to the COMPASS system (https://uwaterloo.ca/compass-system/).

## Notes

**Claire Benny**, PhD

**Brendan T. Smith**, PhD

**Karen A. Patte**, PhD

**Scott T. Leatherdale**, PhD

**Roman Pabayo**, PhD
